# Acknowledgement to reviewers

**DOI:** 10.1530/EO-5-A1

**Published:** 2025-12-16

The editorial board wishes to thank the following individuals who have helped to review manuscripts for *Endocrine Oncology* during 2024. Their assistance is gratefully acknowledged.

**Table:** 

N K Aaronson
A Abbas
M L Abbate
S Adam
S Agosto
N Ahmadi Bidakhvidi
O Alagoz
N Alcala
M Alevizaki
A Aljubran
J Aller
L Al-Mansour
B Altieri
C V Alvarez
V Ambrosini
E Anda Apiñaniz
T E Angell
D Annibali
T Araki
S H Arif
C J Auernhammer

S Backman
S Baldari
C Baldwin
A Bartell
D K Bartsch
S Bazarbashi
J Bell
J S Berek
A Berruti
N Besic
C L Bevan
K Bhaskaran
M W Bishop
L Bodei
L Boldrini
A Bongiovanni
V Boonyaratanakornkit
S A Boorjian
R Brady
P K Brastianos
T Braun
P Breheny
G Brigante
J Brito
L O Buchmann
D Bulzico
P M Bunch
B Burkett
P Burman
L M Butler

M E Cabanillas
A Cagnacci
L Cai
D Capoccia
L Cardoso
C Cardwell
J A Carrasquillo
C Carretero
O Casanovas
O Casar-Borota
R T Casey
R Chammas
D Chan
A Chauhan
L Chen
J Chojnacki
N Chouin
N Chun
A Cistaro
H O D Critchley
J Crona
D Cronin-Fenton
C Cunningham
T Cuny
G Curigliano

A F Daly
A Davies
T Dayton
S De Leo
M I del Olmo-García
M del Rio-Moreno
J Del Rivero
C Deroose
J J Díez
U Dischinger
F Doglietto
H Dralle
S Dream
J A Ducie

P Ear
A Ebbehoj
A Eketunde
M A Elbaset
A-K Elf
T Else
E Estebanez-Perpiña
J Evron

A Faggiano
J A Fagin
P A Fasching
T Felismino
D Ferone
L Filippi
J Flitsch
M C Fragoso
F Frasca

F Gabalec
F Gaia
A C Gao
S Gaujoux
J Gebauer
E P Gelmann
N George
I Gesmundo
K Gewandter
S Gill
L Giovanella
A Glezer
A Glover
J Gómez Tejeda Zañudo
P Goodwin
E Goossens
M Gotthardt
P Goudet
R Graff
C M Grana
D Granberg
G Grani
A B Grossman

M A Habra
J Hallet
C A Hamilton
D E Hansel
N He
A D Herrera Martínez
A L Ho
A O Hoff
L J Hofland
Y Horimoto

A Ibáñez-Costa
A G Ioachimescu
M V Iorio
A M Isidori
K Ito
E Iwabuchi
H Iwase

B Jafar-Mohammadi
M J Jager
J Jiang
C Jimenez
T Juratli
M Justyna Kozieł

P Kadioglu
S S Kakar
G Kaltsas
G A Kaltsas
Y Kanai
I Kareva
D Kastelan
M Kawai
A Kennedy
A A Khan
B Khoo
W G Kim
O Kimpel
O Kiss
C Kitahara
G Klöppel
J Klubo-Gwiezdzinska
A Kondi-Pafiti
A Koneshamoorthy
A L L F Kooi
M Korbonits
J Koshiol
B Kos-Kudła
L P Kowalski
Y N Koyanagi
J Krajewska
M Krausch
I Kreitschmann-Andermahr
M Kroiss
A Kula
J Kwekkeboom

J V Lacey
A Lacroix
J Lado-Abeal
M Laganà
M G E H Lam
B Lecumberri Santamaria
C Lepage
M A Lewis
R Libé
S K Libutti
J Lin
M-F Lin
Y-L Lin
K E Lines
M J Livhits
J J Loonen
C Loucera
P B Loughrey
J P Low

S Mabuchi
A Mafficini
A L Maia
M Marazuela
M Marchioni
M Marino
I Marinoni
A Markou
R C Martin
N Massarweh
A Matrone
H Mazeh
A I McCormack
C Mesguich
A Mohamed
S Mondaca
L Morelli
B Moretti
K Mortezaee
A Munir
R D Murray
A Muth

Y Nagashima
R T Netea-Maier
N Nezami
L Ni
G P Nicolas
M N Nikiforova
T Nohara
G Ntali

J M O’Connor
T Okamoto
J A Overbeek
M Özçelik

K Pacak
F Pakdel
T V Paul
G Pellegriti
A Pera-Rojas
L G Perez-Rivas
P Petrossians
E M Pinto
M Pithukpakorn
G Powathil
J Pozniak
S R Prasad
V Prasad
S Puglisi

D Quelle

R A Ramirez
S N Rao
G Raverot
Y Ren
M D Ringel
S L Rosa
B M Ryan

A Sadanandam
M Sadar
W Saeger
S Saji
L Samalin
K Sasaki
C Schalin-Jantti
F Schmitt
Y Sethi
W Setiawan
M Shakibaei
I Shimon
S B Sidhu
H P Soares
J P Solar Vasconcelos
B Soldevilla
D Sprengers
K Stålberg
S E Steck
M St-Jean
J R Strosberg
Y Sun
K E Sundling
E Świętochowska

J-N Talbot
G Tallini
R Tamimi
G Targher
K Terio
S Todde
E Toledo
Y Tong
T-V-T Tran
G Treglia
M Trevisan
N Tritos
G Trivellin

H Usui

P Valderrabano
N Valdés
C van Kinschot
E van Velsen
T Vandamme
J Vinagre
B Voellger

T Walter
H Wang
W Wang
Z Wang
D Waniczek
F Weber
L E A Wildemberg
K Wolf
J D Wright

B Xing
M Xing
F Xiong

K Yamanoi
C Yang
Y Ye
K Yu
L Yuan

C Zafon
J Zhang
M Zhou

